# The occlusion tests and end-expiratory esophageal pressure: measurements and comparison in controlled and assisted ventilation

**DOI:** 10.1186/s13613-016-0112-1

**Published:** 2016-02-12

**Authors:** Davide Chiumello, Dario Consonni, Silvia Coppola, Sara Froio, Francesco Crimella, Andrea Colombo

**Affiliations:** Dipartimento di Anestesia, Rianimazione (Intensiva e Subintensiva) e Terapia del Dolore, Fondazione IRCCS Ca’ Granda - Ospedale Maggiore Policlinico, Via F. Sforza 35, Milan, Italy; Dipartimento di Fisiopatologia Medico-Chirurgica e dei Trapianti, Università degli Studi di Milano, Milan, Italy; Unità Operativa di Epidemiologia, Fondazione IRCCS Ca’ Granda-Ospedale Maggiore Policlinico, Milan, Italy

**Keywords:** Esophageal pressure, PEEP, ARDS, Transpulmonary pressure, Respiratory mechanics

## Abstract

**Background:**

Esophageal pressure is used as a reliable surrogate of the pleural pressure. It is conventionally measured by an esophageal balloon placed in the lower part of the esophagus. To validate the correct position of the balloon, a positive pressure occlusion test by compressing the thorax during an end-expiratory pause or a Baydur test obtained by occluding the airway during an inspiratory effort is used. An acceptable catheter position is defined when the ratio between the changes in esophageal and airway pressure (∆Pes/∆Paw) is close to unity. Sedation and paralysis could affect the accuracy of esophageal pressure measurements. The aim of this study was to evaluate, in mechanically ventilated patients, the effects of paralysis, two different esophageal balloon positions and two PEEP levels on the ∆Pes/∆Paw ratio measured by the positive pressure occlusion and the Baydur tests and on the end-expiratory esophageal pressure and respiratory mechanics (lung and chest wall).

**Methods:**

Twenty-one intubated and mechanically ventilated patients (mean age 64.8 ± 14.0 years, body mass index 24.2 ± 4.3 kg/m^2^, PaO_2_/FiO_2_ 319.4 ± 117.3 mmHg) were enrolled. In step 1, patients were sedated and paralyzed during volume-controlled ventilation, and in step 2, they were only sedated during pressure support ventilation. In each step, two esophageal balloon positions (middle and low, between 25–30 cm and 40–45 cm from the mouth) and two levels of PEEP (0 and 10 cmH_2_O) were applied. The ∆Pes/∆Paw ratio and end-expiratory esophageal pressure were evaluated.

**Results:**

The ∆Pes/∆Paw ratio was slightly higher (+0.11) with positive occlusion test compared with Baydur’s test. The level of PEEP and the esophageal balloon position did not affect this ratio. The ∆Pes and ∆Paw were significantly related to a correlation coefficient of *r* = 0.984 during the Baydur test and *r* = 0.909 in the positive occlusion test. End-expiratory esophageal pressure was significantly higher in sedated and paralyzed patients compared with sedated patients (+2.47 cmH_2_O) and when esophageal balloon was positioned in the low position (+2.26 cmH_2_O). The esophageal balloon position slightly influenced the lung elastance, while the PEEP reduced the chest wall elastance without affecting the lung and total respiratory system elastance.

**Conclusions:**

Paralysis and balloon position did not clinically affect the measurement of the ∆Pes/∆Paw ratio, while they significantly increased the end-expiratory esophageal pressure.

## Background

For several years now, the esophageal balloon technique has been considered as a reliable surrogate for the pleural pressure measurement [1–3]. However, for different reasons, such as technical issues or difficulty to obtain reliable measurements, this technique was rarely applied in critically ill patients [4]. On the contrary, in the last period of time, with the development of new esophageal balloon catheters which offer good accuracy [5, 6], and several studies showing the utility of this monitoring for ventilator management of ARDS patients [7–9], esophageal pressure has been rediscovered [4, 10].

The most common technique for measuring esophageal pressure employs an esophageal balloon filled with air connected to a long thin catheter [5, 6]. Appropriate filling volumes of air are required to minimize possible errors in the measurement of esophageal pressure [5, 6]. In addition, to limit possible pressure artifacts due to non-homogeneous compression of the esophagus by external structure, it has been suggested to place the esophageal balloon catheter in the lower two-thirds of the esophagus [11]. In spontaneously breathing patients, the conventional test to validate the correct position of esophageal balloon is performed during an end-expiratory occlusion maneuver with the simultaneous measurement of the changes in airway and esophageal pressure during an inspiratory effort (i.e., no air flow) (Baydur’s occlusion test) [12]. The position of the esophageal catheter is considered acceptable when the ratio between the changes in two pressures is close to unity. In healthy subjects with or without anesthesia, when the esophageal catheter was correctly positioned, it has been found that the difference between the changes in esophageal and airway pressure was lower than 15 % [12, 13]. Similarly, Milner et al. [14] found in spontaneous breathing babies that esophageal pressure well correlated with airway pressure supporting the validity of this occlusion test in infants too.

The Baydur occlusion test can be used only in patients with active breathing. In sedated and paralyzed patients, an external manual pressure is applied on the rib cage and the changes in esophageal and airway pressure are recorded during an expiratory pause (positive pressure occlusion test) [4, 15].

In animals, Dechman et al. [16] showed a better relationship between esophageal and airway pressure during the positive pressure occlusion test compared with the Baydur test. The loss of esophagus tension by the administration of neuromuscular block significantly reduced the attenuation of the transmission of pleural pressure through the esophagus. Conversely, in healthy puppies it has not been found any difference in the accuracy between the two occlusion tests [17]. Although both tests are routinely used in critically ill patients, none have assessed how sedation and paralysis, influencing pleural pressure transmission through the esophagus, might affect the accuracy of the esophageal pressure measurements.

The main purpose of this study was to investigate in the same intubated, mechanically ventilated patients the effects of paralysis, two different esophageal balloon positions and two PEEP levels on the ratio between changes in esophageal and airway pressures (∆Pes/∆Paw) recorded by the Baydur and the positive pressure occlusion tests and on the end-expiratory esophageal pressure. In addition, it was also examined how the esophageal balloon position and the PEEP level influenced the partitioned respiratory mechanics (lung and chest wall elastance) in sedated and paralyzed patients.

## Methods

### Study population

The study population consisted of 21 intubated and mechanically ventilated patients admitted to Intensive Care Unit of Fondazione Ca’ Granda Ospedale Maggiore Policlinico, Milan, Italy, after elective surgery or for medical reason, from December 2013 to April 2015. Inclusion criteria were the presence of invasive mechanical ventilation and the absence of contraindications for the placement of an esophageal balloon catheter. Exclusion criteria were patients younger than 18 years, high grade of esophageal varices, severe coagulopathy, recent history of esophageal, gastric or thoracic surgery. The study was approved by the institutional review board of our hospital (www.clinicaltrials.gov NCT02036788), and informed consent was obtained according to the Italian regulations.

### Study protocol

The study protocol consisted of two consecutive parts. In the first part (step one), the patients were deeply sedated and paralyzed. The anesthesia was maintained with infusion of propofol 1 % (200–300 γ/Kg/min) and paralysis with rocuronium (a bolus of 0.8 mg/kg at the begin and subsequently 20 mg every 20 min) to obtain a total myorelaxation. Sedation was titrated to obtain a state equivalent to a Richmond Agitation Sedation Score (RASS) of −5. A tidal volume of 6–8 ml/Kg of predicted body weight was applied during volume-controlled ventilation.

Subsequently in the second part (step two), after about 60 min from the end of the first part, patients were maintained sedated without paralysis and ventilated in pressure support ventilation to insure a tidal volume similar to that of volume-controlled ventilation. Sedation was titrated to obtain a RASS between −3 and −2. Patients were always maintained in supine position at 0° for the entire study. Because the most of the patients were enrolled in intensive care unit after elective general anesthesia (i.e., already sedated and paralyzed), the first and the second parts of the study were not performed in a random fashion and the sedation and paralysis step was always done first. However, the measurements collected in the four different study conditions both during controlled and pressure support ventilation were taken in a random fashion as follows:

1. PEEP 0 cmH_2_O with esophageal balloon placed at 25–30 cm from the mouth (middle position)
2. PEEP 0 cmH_2_O with esophageal balloon placed at 40–45 cm from the mouth (low position)
3. PEEP 10 cmH_2_O with esophageal balloon placed at 25-30 cm from the mouth (middle position)
4. PEEP 10 cmH_2_O with esophageal balloon placed at 40–45 cm from the mouth (low position)

**Fig. 1:**
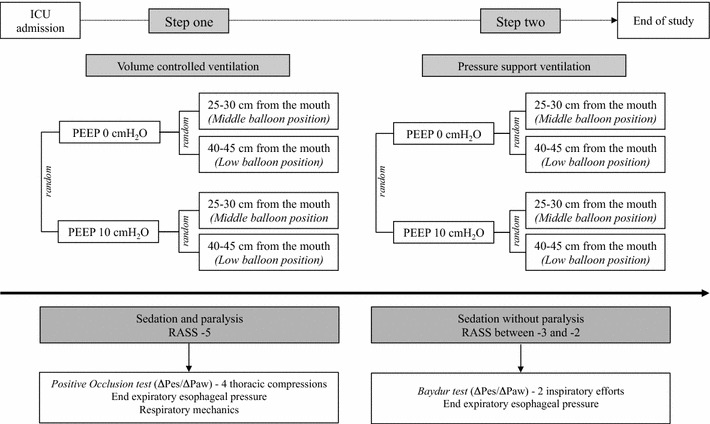
Schematic overview of the study protocol

Two-min recording sections were taken for each condition after a stabilization period (10 min) following the change in PEEP level and esophageal balloon position. A schematic overview of the protocol is shown in Fig. 1.

### Measurements

Airway pressure was measured between the Y piece and the endotracheal tube with a dedicated pressure transducer (MPX 2010 DP, Motorola, Solna, Sweden). Esophageal pressure was measured using a standard balloon catheter (Smart Cath, Viasys, Palm Springs, USA) consisting of a tube 103 cm long with an external diameter of 3 mm and a thin-walled balloon 10 cm long. The gastric and esophageal pressures curves were monitored during the placement of esophageal catheter using the same pressure transducer (MPX 2010 DP, Motorola, Solna, Sweden).

To place the esophageal catheter in the appropriate position for the study, we marked it with tape every 5 cm. Before the insertion in the paralyzed patients, the esophageal catheter was emptied of air and closed with a three-way stopcock. It was introduced transorally and advanced to reach the stomach (generally at a least depth of 55 cm from the mouth). It was inflated with 1.5 ml of air. Depending on the tension of the catheter, the angle in the mouth and height of the patient, the gastric position was found between 50 and 55 cm from the mouth within a range of 5 cm. The intragastric position of the catheter was confirmed by a rise in intra-abdominal pressure following external manual epigastric compression. Then, it was retracted into the esophagus (i.e., confirmed by the presence of cardiac artifacts in the pressure tracing and by the difference in the absolute pressure), at a distance between 40 and 45 cm from the mouth that was the “low position.” The “middle position” was found retracting the esophageal catheter of 15 cm from this point (25–30 cm from the mouth). It was randomly placed in the low or middle esophageal position. The amount of gas in the balloon was periodically checked throughout the experiment.

Esophageal pressure when located in the appropriate position and airway pressure were recorded and processed, sampled at 100 Hz, on a dedicated data acquisition system (Colligo and Computo, www.elekton.it).

### Positive pressure occlusion test

In sedated and paralyzed patient, the test was performed by applying four manual thoracic compressions during end-expiratory holds while simultaneously recording the esophageal and airway pressure. The ∆Pes/∆Paw ratio was calculated as the ratio between maximum (positive) changes in esophageal and airway pressure.

Four measurements of ∆Pes/∆Paw ratio were recorded in each of the four study conditions.

### Baydur occlusion test

During pressure support ventilation, the test was performed during an expiratory hold and similarly to positive pressure occlusion test, by recording the esophageal and airway pressure. Similarly, the ∆Pes/∆Paw ratio was calculated as the ratio between maximum (negative) changes in esophageal and airway pressure during two inspiratory efforts. Two measurements of ∆Pes/∆Paw ratio were recorded in each of the four study conditions.

### End-expiratory esophageal pressure

The end-expiratory esophageal pressure was measured during an end-expiratory pause in sedated and paralyzed patients and during an end-expiratory pause just before an inspiratory effort in sedated patients. It was measured twice in each of the four study conditions.

### Respiratory mechanics

In sedated and paralyzed patients, during controlled mechanical ventilation the static airway and esophageal pressure were measured during an end-inspiratory and end-expiratory pause. Only in sedated and paralyzed patients, elastance-derived end-inspiratory transpulmonary pressure, respiratory system and lung and chest wall elastance were computed according to the following formula [18]:

- Elastance-derived end-inspiratory transpulmonary pressure: (airway pressure during an inspiratory pause × lung elastance/respiratory system elastance)
- Respiratory system elastance (Ers) (cmH_2_O/L) = (airway pressure during an inspiratory pause − airway pressure at PEEP during an expiratory pause)/tidal volume
- Lung elastance (El) (cmH_2_O/L) = (transpulmonary pressure during an inspiratory pause − transpulmonary pressure at PEEP during an expiratory pause/tidal volume
- Chest wall elastance (Ecw) (cmH_2_O/L) = (esophageal pressure at end inspiration − esophageal pressure at PEEP)/tidal volume

For each of the four study conditions, respiratory mechanics measurements were taken twice during two different end-inspiratory pauses and two different end-expiratory pauses.

### Statistical analysis

Given that study outcomes (∆Pes/∆Paw ratio, end-expiratory esophageal pressure, respiratory mechanics measurements) have been measured several times in each patient, to take into account intra-patient correlation we used a multiple linear random-intercept regression models (Rabe-Hesketh S, Skrondal A. Multilevel and Longitudinal Modeling Using Stata, 2nd Edition. Stata Press 2008) to investigate the effects of the variables PEEP (0 vs 10 cmH_2_O), esophageal balloon position (low vs middle) and paralysis (with or without).

We included, in the regression models, the main effects and their first- and second-order interaction terms. In addition, measurements were repeated in each of four study conditions: four ∆Pes/∆Paw ratio measurements in paralyzed patients and two in non-paralyzed patients; two end-expiratory esophageal pressure measurements in both paralyzed and non-paralyzed patients; two respiratory mechanics measurements (partitioned elastance and elastance-derived end-inspiratory transpulmonary pressure) in paralyzed patients only. The intra-patients repeatability of these measurements was evaluated by calculating the intra-class correlation coefficient (ICC) with the same random-intercept models including first- and second-order interaction terms.

Statistical analysis was performed with Stata 13 (StataCorp. 2013. Stata: Release 13. Statistical Software. College Station, TX: StataCorp LP).

## Results

The baseline characteristics of the enrolled patients are reported in Table 1.

**Table 1:** Main characteristics of the study population at enrollment

Patient’s characteristics	Overall population, *n* 21
Age, years	64.8 ± 14.0
Sex, *n*° male (%)	13 (62)
Height, cm	170 ± 12
Weight, kg	70.6 ± 14.9
BMI, kg/m^2^	24.2 ± 4.3
PEEP, cmH_2_O	6.6 ± 2.2
Airway plateau pressure, cmH_2_O	17.2 ± 4.9
PaO_2_/FiO_2_	319.4 ± 117.3
FiO_2_	0.4 ± 0.1
PaCO_2_ mmHg	40.7 ± 6.2
Elastance_RS_, cmH_2_O/L	19.0 ± 6.5
SAPS II	36.2 ± 15.3
Diagnosis, *n*° (%)	
Abdominal surgery	7 (34)
Urologic surgery	4 (19)
Vascular surgery	3 (14)
Postanoxic coma	3 (14)
Other	4 (19)

Data are presented as mean ± standard deviation unless indicated otherwise

*BMI* body mass index, *PEEP* positive end-expiratory pressure, *PaO*_*2*_ arterial partial pressure of oxygen, *PaCO*_*2*_ arterial partial pressure of carbon dioxide, *PaO*_*2*_ */FiO*_*2*_ the ratio of arterial oxygen partial pressure to fractional inspired oxygen, *FiO*_*2*_ fractional inspired oxygen, *SAPS* Simplified Acute Physiology Score, *Elastance*_*RS*_ elastance of respiratory system, *n*° number

### Esophageal and airway pressure ratio

During the positive pressure occlusion test, the pressure manually applied on the rib cage generated average positive changes in esophageal pressure of 6.9 ± 2.0 cmH_2_O and of 7.0 ± 2.0 cmH_2_O at 0 and 10 cmH_2_O of PEEP respectively, while during the Baydur test the inspiratory muscle generated average negative changes in esophageal pressure of 7.5 ± 4.4 cmH_2_O and of 7.3 ± 4.7 cmH_2_O at 0 and 10 cmH_2_O of PEEP. Paralysis did not affect the changes in esophageal pressure at 0 and 10 cmH_2_O of PEEP (*p* = 0.325).

**Fig. 2:**
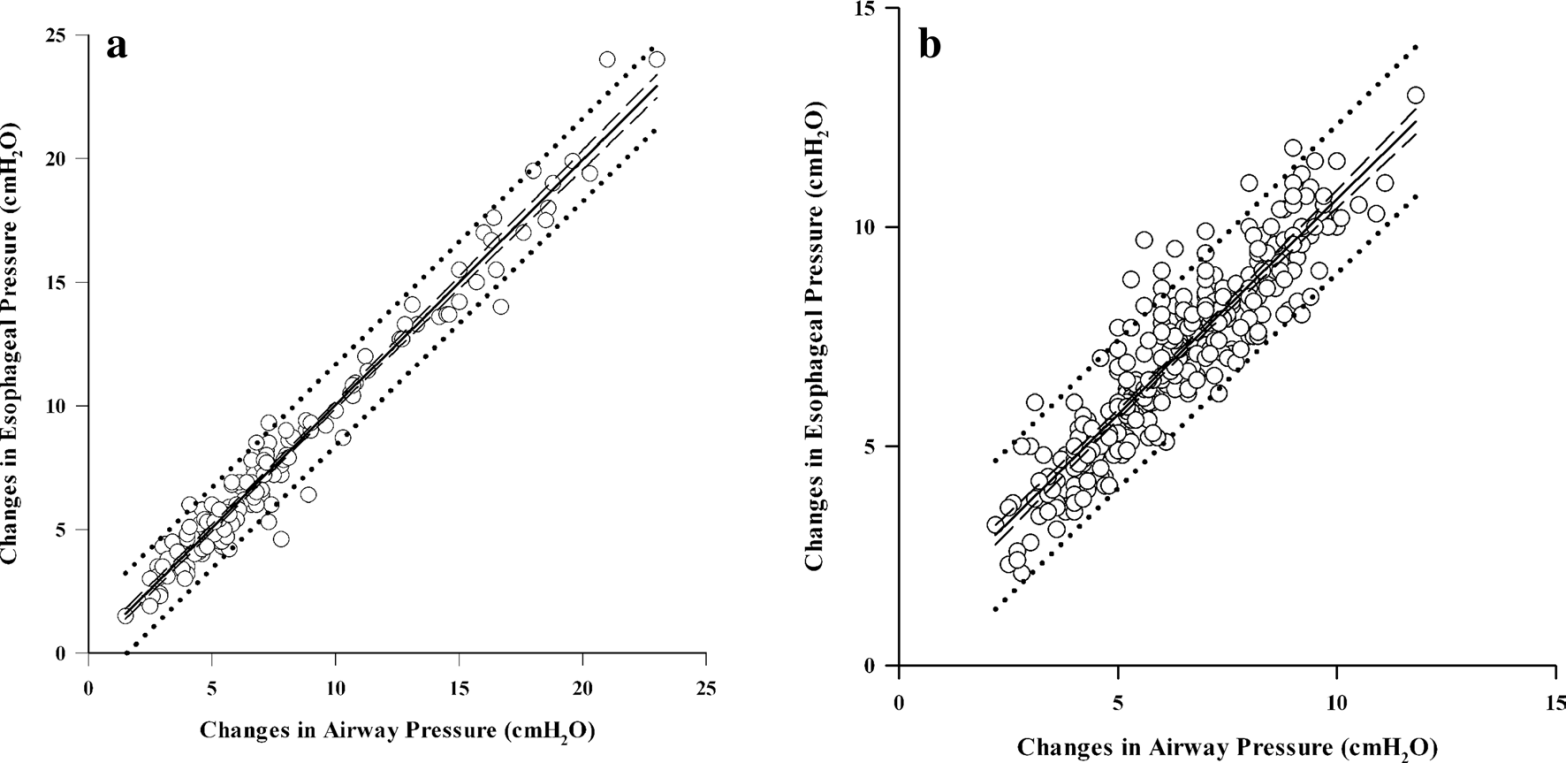
Linear regression between the changes in airway pressure (*x* axis) (absolute terms) and esophageal pressure (*y* axis); **a** Baydur occlusion test, *y* = 0.096 + (0.993 × *x*), *r* = 0.984; **b** positive pressure occlusion test, *y* = 0.818 + (0.982 × *x*), *r* = 0.909. In each graph, *continuous line* represents the regression line, *dashed lines* represent 95 % CI bounds, and *dotted lines* represent 95 % prediction interval bounds. *Empty dots* represent each single ∆Pes/∆Paw ratio obtained in all enrolled patients

On average, the ∆Pes/∆Paw ratio was significantly higher in paralyzed (+0.11) compared with non-paralyzed patients (Table 2). Different from the presence of paralysis, the level of PEEP and the esophageal balloon position did not influence ∆Pes/∆Paw ratio as demonstrated in Table 2 (paralysis versus no paralysis *p* < 0.001; PEEP 10 vs PEEP 0 *p* = 0.376; low vs middle esophageal balloon position *p* = 0.515). The changes in esophageal and airway pressure were significantly related to a correlation coefficient of *r* = 0.984 and of *r* = 0.909 during the Baydur and the positive pressure occlusion tests, respectively (Fig. 2).

**Table 2:** Effect of paralysis, PEEP and esophageal balloon position on the ratio between changes in esophageal pressure and airway pressure (ΔPes/ΔPaw)

ΔPes/ΔPaw^a^			Statistical analysis^b^				
*PEEP* (cmH_2_O)	Middle balloon position	Low balloon position		Coeff.	95 % CI		*p*
Positive pressure occlusion test—*P*			P versus no P	0.11	0.06	0.17	*<0.001*
0	1.14 ± 0.18	1.12 ± 0.15	PEEP 10 versus 0	−0.03	−0.09	0.03	0.376
10	1.09 ± 0.17	1.15 ± 0.14	Low versus middle position	−0.02	−0.08	0.04	0.515
Baydur Occlusion test—*no P*			P × low position	0.01	−0.07	0.08	0.848
0	1.02 ± 0.16	1.00 ± 0.13	P × PEEP 10	−0.02	−0.09	0.06	0.638
10	1.00 ± 0.11	1.05 ± 0.13	Low position × PEEP 10	0.08	−0.01	0.16	0.084
			P × Low position × PEEP 10	−0.01	−0.12	0.10	0.849

Multiple linear random-intercept regression models including main effects and interaction terms

“P versus no P” (paralysis versus no paralysis) means a comparison between Baydur and positive pressure occlusion tests

Statistically significant p value is in italics

*ΔPes* change in esophageal pressure, *ΔPaw* change in airway pressure, *PEEP* positive end-expiratory pressure (cmH_2_O), *P* paralysis, *no P* no paralysis

Data are presented as ^a^mean ± standard deviation and as ^b^ regression coefficient with 95 % confidence interval

### End-expiratory esophageal pressure

The end-expiratory esophageal pressure was significantly influenced by paralysis, balloon position and level of PEEP. In particular, it was significantly higher in paralyzed patients compared with sedated patients (+2.47 cmH_2_O), when esophageal balloon was positioned in the low position (+2.26 cmH_2_O) and when level of PEEP was 10 compared to PEEP 0 (+4.01 cmH_2_O), as demonstrated by the positive regression coefficients (Table 3). We found two significant interactions: P × Low Position (Paralysis × Low esophageal balloon position *p* < 0.001) and P × PEEP 10 (Paralysis × PEEP 10 *p* < 0.001). However, both the regression coefficients were negative (−2.3 and −2, respectively), indicating that the effects of low balloon esophageal position in paralyzed patients as well as the effect of PEEP 10 in paralyzed patients do not sum each other (Table 3).

**Table 3:** Effect of paralysis, PEEP and esophageal balloon position on end-expiratory esophageal pressure (cmH_2_O)

End-expiratory esophageal pressure^a^			Statistical analysis^b^				
PEEP (cmH_2_O)	Middle balloon position	Low balloon position		Coef.	95 % CI		*p*
Sedation with paralysis—*P*			P versus no P	2.47	1.66	3.27	*<0.001*
0	10.1 ± 2.8	10.1 ± 2.8	PEEP 10 versus 0	4.01	3.21	4.81	*<0.001*
10	12.2 ± 2.1	13.3 ± 2.2	Low versus middle position	2.26	1.46	3.05	*<0.001*
Sedation without paralysis—*no P*			P × Low position	−2.30	−3.43	−1.16	*<0.001*
0	7.7 ± 3.5	9.9 ± 3.7	P × PEEP 10	−2.00	−3.12	−0.87	*0.001*
10	11.7 ± 3.6	13.6 ± 3.1	Low position × PEEP 10	−0.31	−1.43	0.80	0.584
			P × Low position × PEEP 10	1.47	−0.11	3.06	0.069

Multiple linear random-intercept regression models including main effects and interaction terms

Statistically significant p values are in italics

*PEEP* positive end-expiratory pressure, *P* paralysis, *no P* no paralysis

Data are presented as ^a^mean ± standard deviation and as ^b^ regression coefficient with 95 % CI

### Respiratory mechanics

In sedated and paralyzed patients, the lung elastance was slightly lower when computed with the esophageal balloon in the low position compared with middle position (−0.99) which is statistically different but clinically insignificant (Table 4).

**Table 4:** Effect of PEEP and esophageal balloon position on partitioned respiratory mechanics in sedated and paralyzed patients

Respiratory mechanics^a^			Statistical analysis^b^				
PEEP (cmH_2_O)	Middle balloon position	Low balloon position		Coef.	95 % CI		*p*
Elastance_RS_ cmH_2_O/L			PEEP 10 versus 0	−0.06	−0.75	0.62	0.857
0	18.8 ± 5.1	18.3 ± 4.9	Low versus middle position	−0.57	−1.26	0.11	0.103
10	18.8 ± 5.1	18.5 ± 5.1	Low position × PEEP 10	0.29	−0.68	1.26	0.559
Elastance_L_ cmH_2_O/L							
0	12.5 ± 5.1	11.6 ± 4.7	PEEP 10 versus 0	0.64	−0.26	1.55	0.164
10	13.2 ± 4.1	13.3 ± 4.2	Low versus middle position	−0.99	−1.90	−0.08	*0.033*
			Low position × PEEP 10	1.13	−0.15	2.42	0.083
Elastance_CW_ cmH_2_O/L							
0	6.3 ± 3.4	6.7 ± 4.2	PEEP 10 versus 0	−0.71	−1.39	−0.02	*0.044*
10	5.6 ± 2.7	5.2 ± 3.0	Low versus middle position	0.42	−0.27	1.11	0.231
			Low position × PEEP 10	−0.85	−1.81	0.12	0.088
El–End–Insp Tp cmH_2_O							
0	6.3 ± 2.6	5.7 ± 2.2	PEEP 10 versus 0	8.23	7.76	8.70	*<0.001*
10	14.6 ± 2.7	13.5 ± 2.5	Low versus middle position	−0.61	−1.09	−0.14	*0.011*
			Low position × PEEP 10	−0.50	−1.17	0.17	0.142

Statistically significant p values are in italics

*PEEP* positive end-expiratory pressure, *Elastance*_*RS*_ elastance of respiratory system, *Elastance*_*L*_ elastance of lung, *Elastance*_*CW*_ elastance of chest wall, *El–End–Insp Tp* elastance-derived end-inspiratory transpulmonary pressure

Data are presented as ^a^mean ± standard deviation. ^b^ *p* value from linear random-intercept regression models

Elastance-derived end-inspiratory transpulmonary pressure was significantly higher when estimated with the esophageal balloon located in the middle position at both level of PEEP (Table 4).

At the two balloon positions, the increase in PEEP significantly reduced the chest wall elastance (−0.71) without affecting the lung and total respiratory system elastance and increased the elastance-derived end-inspiratory transpulmonary pressure (+8.23) (Table 4).

### Intra-patients repeatability measurements

Repeatability of measurements was low both for the Baydur occlusion test (ICC = 0.13) and for positive pressure occlusion test (ICC = 0.16), but it was high for end-expiratory esophageal pressure (ICC = 0.62), elastance of respiratory system (ICC = 0.90), elastance of lung (ICC = 0.78), elastance of chest wall (ICC = 0.77) and elastance-derived end-inspiratory transpulmonary pressure (ICC = 0.77).

## Discussion

The primary findings of this study are: (1) the ∆Pes/∆Paw ratio was faintly different when computed with the Baydur and the positive pressure occlusion tests; (2) end-expiratory esophageal pressure was higher in sedated and paralyzed patients, with the esophageal balloon in the low position and with higher PEEP level; and (3) lung elastance was slightly lower when computed with the esophageal balloon in the low position while the PEEP reduced the chest wall elastance without affecting the lung and respiratory system elastance.

The estimation of respiratory mechanics, lung stress and muscle activity requires the measurement of transpulmonary pressure, calculated as the difference between airway and pleural pressure. Due to the impossibility of directly measuring pleural pressure in clinical practice, esophageal pressure has been proposed as a reliable surrogate [1–3]. Although the absolute values of esophageal pressure are not equal to the absolute values of pleural pressure, the difference between the changes in intrathoracic and esophageal pressure was found to be in the order of 1 % [19] and mainly depends on the elastance of the esophageal walls, on the surrounding structures and on the mechanical properties of esophageal balloon [5, 6, 11, 19]. Since the earliest animals and human studies, pleural pressure has usually been estimated indirectly from an esophageal catheter equipped by a balloon filled by air [19, 20]. Besides the appropriate air filling, a correct position of the balloon in the esophagus is fundamental to obtain an accurate estimation of the pleural pressure [4, 10, 12].

In spontaneously breathing subjects, the “optimal position” of the esophageal balloon catheter is usually found by applying the “Baydur occlusion test” [4]. With this test in a small group of not intubated patients, it was found that the average ratio between changes in esophageal and airway pressure was 0.90 ± 0.40 with individual data ranging from 0.61 to 1.10 [12]. In non-paralyzed anesthetized subjects, with the balloon optimally located, this ratio amounted to 0.98 ± 0.03 [13].

However, due to the impossibility to perform the “Baydur” test in deeply sedated or paralyzed patient, a positive pressure occlusion test has been proposed [4, 15].

Sedation and paralysis may affect the transmission of pleural pressure through the esophageal wall altering the validity of the positive pressure occlusion test compared with the “Baydur” test [21].

In the present study, we found that the ∆Pes/∆Paw ratio was significantly higher (+0.11) when calculated with positive occlusion test compared with Baydur’s test (paralysis vs no paralysis). During an occlusion test, either by compressing the thorax or by generating inspiratory efforts against a closed airway, there are actually slight changes in the lung volume and transpulmonary pressure due to the compressibility of intrathoracic gas, rather than constant. It could be also possible to have a slightly higher ∆Pes/∆Paw ratio obtained by compressing the thorax with gas compression, than the one obtained by Baydur’s maneuver with gas expansion. However, from the clinical viewpoint this difference is negligible. Contrary to dogs in which the ratio between esophageal and airway pressure was better during positive occlusion test compared with standard occlusion test, probably because the esophagus is composed of entirely striated muscle, in humans the middle and distal esophageal body is mainly constituted by smooth muscle with a much lower influence of sedative drugs and neuromuscular blockers [22–25]. This was also confirmed by Hedenstierna et al. [26] which showed no difference in the esophageal elastance (i.e., similar elasticity) between awake and anesthetized subjects. Furthermore, the higher level of sedation and the neuromuscular block which could decrease the lung volume did not affect the validity of the two tests.

In our study, ∆Pes/∆Paw ratio recorded by the Baydur and the positive pressure occlusion tests was not affected neither by the PEEP level nor by the esophageal balloon position.

Similar data were reported in healthy subjects in whom the changes in esophageal and airway pressure differed less than 10 % at different lung volumes, over the range of vital capacity [27] and with the esophageal balloon in different positions (middle and low) [12].

Based on the whole data, the Baydur and the positive pressure occlusion tests can be considered similar, suggesting that clinicians do not have to repeat the test or reposition the esophageal catheter when the subject is paralyzed or, on the contrary, is waked up.

Although in our study a considerable number of measurements of ∆Pes/∆Paw ratio showed a standard deviation ranged from 0.11 to 0.18, which can be due to intra-patient differences, differences between the four manual thoracic compressions or between the two inspiratory efforts intra- and inter-patients, all the ratios are close to unity and according to us the differences were not clinically relevant, including the effect of paralysis. Furthermore, all the explored interactions were not statistically significant. Therefore, our results showed that there is not a better condition to obtain a better match between ∆Pes and ∆Paw, and in fact, we consider ∆Pes/∆Paw ratio as adequate in every explored condition.

The low intra-patient repeatability could be due to differences in the manual thoracic compressions performed by the principal investigator and in the respiratory efforts performed by the patient; however, thank to these differences we explored a wide range of generated airway (from 1.5 to 23 cmH_2_O) and esophageal pressures (from 1.5 to 24 cmH_2_O). Within this wide range of pressures, the changes in esophageal and airway pressure were significantly related to a correlation coefficient of *r* = 0.984 and of *r* = 0.909 during the Baydur and the positive pressure occlusion tests, respectively.

Although questionable, the use of the end-expiratory esophageal pressure, considering the results of several recent studies based on the esophageal pressure to titrate PEEP [7, 28, 29], should at least require a correct measurement [21]. In this study, end-expiratory esophageal pressure was significantly higher when the balloon was placed in the low position, in deeply sedated and paralyzed patients. It was also influenced by the PEEP level confirming the results of previous studies [7, 30].

When the esophageal balloon is in the middle part of the esophagus, it is mainly influenced by the weight of the heart, while in the low position by the weight of the heart plus the lung. Furthermore, the loss of muscle tone can increase the gravitational weight of these structures over the esophagus. Contrary to the ∆Pes/∆Paw ratio, the end-expiratory esophageal pressure is clearly influenced by the balloon position and paralysis. Although previous studies have shown the possible utility of setting PEEP according to the static end-expiratory esophageal pressure for minimizing the alveolar collapse [7, 30], the end-expiratory esophageal pressure was not found to be related to the lung weight or chest wall elastance [18]. The end-expiratory esophageal pressure can be significantly higher in the presence of neuromuscular paralysis, in higher PEEP level and when esophageal balloon is placed in the low esophagus (Table 3). In addition, the static end-expiratory esophageal pressure has proved to be influenced by the elastic recoil of the balloon and of the esophagus and by the surrounding structures [10, 11, 31–33]. According to these artifacts, several correction factors have been introduced [28, 30]. However, the use of static end-expiratory esophageal pressure can be reasonable in the same clinical condition (PEEP, balloon position, paralysis) to evaluate the clinical changes over time.

The partitioned respiratory mechanics (lung and chest wall) and end-inspiratory transpulmonary pressure are often calculated in patients with acute respiratory failure to evaluate the effects of different tidal volumes or PEEP as a means of minimizing stress and strain and the ventilation-induced lung injury. To avoid any possible artifacts due to the presence of muscle activity, in the current study the respiratory mechanics were measured only in sedated and paralyzed patients. Elastance-derived end-inspiratory transpulmonary pressure and lung elastance were only minimally affected by the position of the esophageal balloon, and similarly to the results of the ∆Pes/∆Paw ratio, they can be considered independently from the position of the balloon. In addition, the PEEP only reduced the chest wall elastance without affecting the lung and total respiratory system elastance and, as expected, increased the elastance-derived end-inspiratory pressure.

## Limitations

Only one type of a traditional air-filled balloon catheter with a fixed amount of air was evaluated. Nevertheless, these findings can still be translated to other types of esophageal catheters if the recommended amount of air is used to inflate the balloon [5, 6]. Liquid-filled catheters, despite presenting possible benefits compared with air-filled, were not examined for the lack of a commercially available device and for the possible higher amount of cardiac artifacts [34, 35]. We did not measure the level of paralysis of our patients; however, all patients were completely paralyzed during the first step of our study as demonstrated by the absence of any inspiratory/expiratory effort during the occlusion maneuver and were able to provide spontaneous respiratory efforts against a closed airway in the second step of the study. The study was conducted in mainly postoperative patients without acute respiratory failure with relatively normal respiratory system compliance. Therefore, our findings should be confirmed in patients suffering from severe respiratory failure with reduced respiratory system compliance.

## Conclusions

In mechanically ventilated patients, the ∆Pes/∆Paw ratio recorded by the positive occlusion test and the Baydur test was not affected neither by the PEEP level nor by the esophageal balloon position, while it was slightly affected by the paralysis. The paralysis, the balloon placed in the low position and higher level of PEEP increased the end-expiratory esophageal pressure.

## Abbreviations

PEEP: positive end-expiratory pressure
ARDS: acute respiratory distress syndrome
El: lung elastance
Ers: respiratory system elastance
Ecw: chest wall elastance
El-End-Insp Tp: elastance-derived end-inspiratory transpulmonary pressure
ICC: intra-class correlation coefficient
BMI: body mass index
PaO_2_: arterial partial pressure of oxygen
PaCO_2_: arterial partial pressure of carbon dioxide
PaO_2_/FiO_2_: the ratio of arterial oxygen partial pressure to fractional inspired oxygen
FiO_2_: fractional inspired oxygen
SAPS: Simplified Acute Physiology Score
ΔPes: change in esophageal pressure
ΔPaw: change in airway pressure
